# Hepatocellular carcinoma: recent advances and emerging medical therapies

**DOI:** 10.12688/f1000research.24543.1

**Published:** 2020-06-17

**Authors:** Kwan-Lung Ko, Lung-Yi Mak, Ka-Shing Cheung, Man-Fung Yuen

**Affiliations:** 1 Department of Medicine, The University of Hong Kong, Queen Mary Hospital, Hong Kong, Hong Kong; 2 State Key Laboratory of Liver Research, The University of Hong Kong, Hong Kong, Hong Kong

**Keywords:** hepatocellular carcinoma, medical therapy

## Abstract

Hepatocellular carcinoma remains a deadly disease with poor prognosis in patients with unresectable cancer. Trans-arterial chemoembolization is the primary locoregional therapy for intermediate-stage hepatocellular carcinoma, with an estimated median overall survival of less than two years. For almost a decade, sorafenib has been the only standard systemic treatment for metastatic disease or tumors which progress or are considered unsuitable for locoregional therapy. Major breakthroughs have been made over the past few years in the management of hepatocellular carcinoma, especially in medical therapies for advanced disease. In this article, recent advances in intra-arterial therapy, multi-kinase inhibitors, and immunotherapy will be reviewed.

## Introduction

Among all cancers globally, liver cancer ranked the sixth in incidence and the fourth in mortality, accounting for 841,080 new cases and 781,631 deaths worldwide in 2018 ^1^. Hepatocellular carcinoma (HCC) is the predominant form of primary liver cancer, with risk factors including hepatitis B virus (HBV) and hepatitis C virus (HCV) infection as well as cirrhosis of any etiology such as alcoholic liver disease, non-alcoholic steatohepatitis, and primary biliary cholangitis. Diagnosis of HCC is usually made by contrast enhanced imaging. Barcelona Clinic Liver Cancer (BCLC) staging remains the most commonly used staging system ^2, 3^. BCLC staging incorporates tumor burden, liver reserve, and performance status and classifies HCC into very early stage (0), early stage (A), intermediate stage (B), advanced stage (C), and terminal stage (D) ^4^. The management strategy can then be recommended according to different stages of disease ^2^. Recently, a popular staging system, the Hong Kong liver cancer staging system (HKLC), which is derived from a group of Hong Kong experts ^5^, was introduced. According to this study, it has a better discriminating power than BCLC ^6^. Nevertheless, it is noteworthy that BCLC is derived from mostly HCV-related HCC cases, while HKLC is derived from an Asian cohort where HBV is the predominant risk factor for HCC. Surgical resection, tumor ablation, and liver transplantation are curative treatments indicated for very early and early stage HCC. Owing to a lack of widely implemented HCC surveillance programs in many parts of the world, most patients are unfortunately diagnosed at an advanced stage, precluding curative therapy ^7^.

Non-surgical management for HCC includes locoregional intra-arterial therapy, systemic treatment with multi-kinase inhibitors, and immunotherapy. Recent advances and emerging strategies in these modalities to improve the outcome of HCC will be reviewed.

## Intra-arterial therapy

### Trans-arterial chemoembolization

Trans-arterial chemoembolization (TACE) is the most popular form of intra-arterial therapy and is the established first-line treatment for intermediate-stage HCC ^2, 3^. It involves arterial catheterization, usually via the femoral artery, to cannulate the hepatic arterial branches. Cytotoxic agent, usually doxorubicin or cisplatin, is then delivered to the highly vascularized liver tumor. It is followed by blocking of the feeding vessel with an embolization agent, commonly lipiodol, to induce ischemic necrosis of the tumor. The rest of the liver parenchyma is relatively preserved as a result of dual blood supply from both the portal vein and the hepatic artery ^8^. Significant improvement of survival was shown in patients with locally advanced HCC and Child-Pugh A or B cirrhosis treated with TACE as compared with best supportive care, with relative risk of death reduction more than 50% ^9– 11^ ( Table 1). Post-embolization syndrome, liver failure due to ischemic damage, biliary injury, gastroduodenal ulceration, and renal dysfunction are potential complications of TACE. Acute liver decompensation may occur in up to one-fifth of patients undergoing TACE ^12^. Dosage of cytotoxic agent, baseline liver function as reflected by bilirubin and prothrombin time, and stage of cirrhosis are identified risk factors. Absolute contraindications to TACE include decompensated cirrhosis, renal failure, main portal vein obstruction, extensive tumor burden, and technical difficulties ^13^. Despite gradual improvement in efficacy and survival, patient selection for the initiation of TACE, the evaluation of tumor response, and the decision on the frequency and duration of therapy are not standardized because of marked heterogeneity within the group of intermediate-stage HCC ^14^. The Hepatoma Arterial-Embolization Prognostic (HAP) score, Assessment for Retreatment with TACE (ART) score, and selection for TACE treatment (STATE) score are prognostic scores derived from combinations of tumor diameter, radiological response, alpha-fetoprotein (AFP) level, and liver biochemistry (bilirubin, aspartate aminotransferase [AST], and C-reactive protein) to better stratify and select patients for the initiation of or subsequent TACE ^15– 18^. However, controlled studies are required to determine whether implementing the prognostic scores in addition to BCLC staging can further improve treatment response and survival in patients receiving TACE. In addition, better definition of TACE failure is also needed, as it correlates with patient survival and mandates switching TACE to an alternative treatment strategy.

**Table 1.** Trials on intra-arterial therapies for hepatocellular carcinoma.

	Study arm	Control arm	No. of patients	Outcome	Major side effects for study arm
Llovet *et al*. ^9^	Embolization arm: gelatin sponge Chemoembolization arm: gelatin sponge plus doxorubicin	Placebo	112	Mean survival 25.3 versus 28.7 versus 17.9 months; HR 0.47, 95% CI 0.25–0.91, *P* = 0.025 for chemoembolization versus placebo	Cholecystitis, leucopenia, ischemic biliary and liver injury, infection, allergic dermatitis, alopecia
Lo *et al*. ^10^	TACE	Placebo	79	Estimated 1-, 2-, and 3-year survival 57%, 31%, and 26% versus 32%, 11%, and 3% RR of death 0.50, 95% CI 0.31–0.81, *P* = 0.005	Fever, abdominal pain, vomiting, ascites
Yuen *et al*. ^11^	TACE	Conservative care	96	Mean survival 31.2 versus 14.1 months, *P* = 0.0126	Hepatic decompensation
Lammer *et al*. ^19^	DEB-TACE	TACE	212	Tumor response at 6 months 51.6% versus 43.5%, *P* = 0.110 for superiority	Significant reduction in liver toxicity and doxorubicin-related side effects in DEB-TACE arm
Salem *et al*. ^21^	Y90 radioembolization	TACE	179	Time to progression >26 versus 6.8 months, *P* = 0.001	Less diarrhea and hypoalbuminemia in Y90 arm
Kudo *et al*. ^22^	TACE plus sorafenib	TACE	156	Median PFS 25.2 versus 13.5 months, *P* = 0.006 Median TTUP 26.7 versus 20.6 months, *P* = 0.020	Thrombocytopenia, hand-foot- skin reaction, hypertension, increased lipase and amylase, neutropenia, fatigue, diarrhea, erythema multiforme more common in study arm
Ricke *et al*. ^23^	SIRT plus sorafenib	Sorafenib	424	Median survival 12.1 versus 11.4 months; HR 1.01, 95% CI 0.81–1.25, *P* = 0.953	Hyperbilirubinemia and fatigue more common in study arm

CI, confidence interval; DEB-TACE, TACE with drug-eluting beads; HR, hazard ratio; PFS, progression-free survival; RR, relative risk; TACE, trans-arterial chemoembolization; TTUP, time to TACE untreatable progression; SIRT, selective internal radiation therapy; Y90, yttrium-90.

### Other forms of intra-arterial therapy

TACE with drug-eluting beads (DEB-TACE) and trans-arterial radio-embolization (TARE) are alternative intra-arterial locoregional therapies for HCC ( Table 1). In DEB-TACE, cytotoxin-carrying microspheres are used instead of lipiodol, allowing simultaneous delivery of chemotherapeutic and embolization agents. The potential advantage of DEB-TACE is more sustained and selective drug delivery to the tumor with less systemic absorption and toxicity. However, evidence thus far does not show a clear benefit of DEB-TACE over conventional TACE. The PRECISION V trial is a phase II randomized trial comparing tumor response between DEB-TACE and TACE in 212 patients with unresectable HCC. Superiority in the primary endpoint of tumor response rate at 6 months was not met (51.6% for DEB-TACE versus 43.5% for TACE, *P* = 0.110 for superiority), but a significant reduction in liver toxicity and doxorubicin-related side effects was seen in the DEB-TACE arm ^19^. TARE is a form of selective internal radiation therapy (SIRT), in which radioactive Yttrium-90 (Y90) microspheres are introduced into the tumor vasculature. The main anti-tumor effect in TARE is achieved by radiation instead of embolization. As the patency of the hepatic artery is maintained, TARE can be used in HCC with main portal vein invasion or thrombosis, which is considered a contraindication for conventional TACE. A meta-analysis of eight studies involving 1,500 patients demonstrated superiority of TARE over TACE in overall survival (OS), 3-year OS, time to progression (TTP), and hospitalization days ^20^. A subsequent phase II randomized trial also showed significantly longer TTP in the TARE group compared with conventional TACE (>26 months versus 6.8 months, *P* <0.01) ^21^. However, improvement of OS was not shown. A randomized controlled trial comparing DEB-TACE and TARE is currently underway (NCT01381211).

## Multi-kinase inhibitors

**Figure 1.**
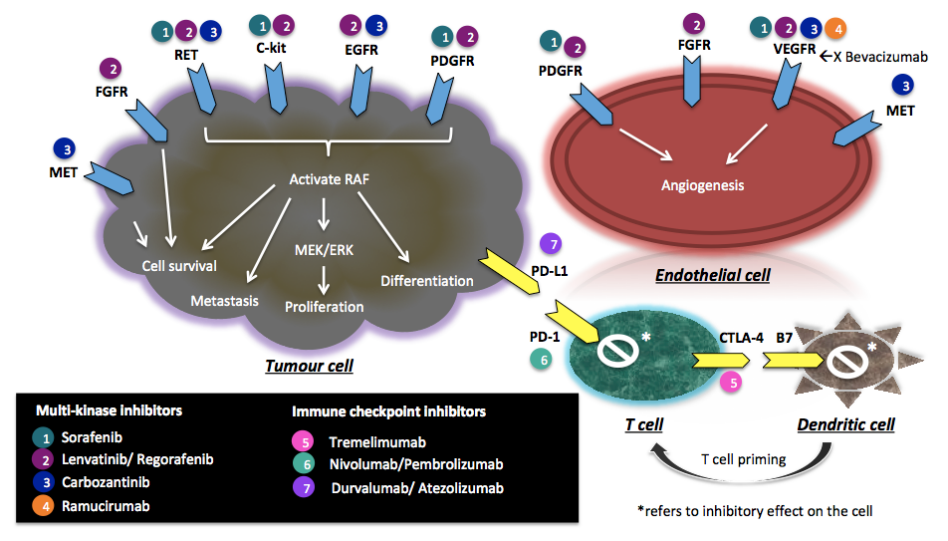
Potential treatment targets for systemic therapy in hepatocellular carcinoma. CTLA-4, cytotoxic T-lymphocyte-associated antigen 4; ERK, extracellular-signal-regulated kinase; FGFR, fibroblast growth factor receptor; MEK, mitogen-activated protein kinase/ERK kinase; PD-1, programmed cell death protein 1; PDGFR, platelet-derived growth factor receptor; PD-L1, programmed death ligand-1; RET, rearrange during transfection; VEGFR, vascular endothelial growth factor receptor.

Tumor cell proliferation, differentiation, and angiogenesis are postulated to be mediated by multiple intracellular and cell surface protein kinases with their downstream pathways, as depicted in Figure 1 ^24^. Sorafenib, an inhibitor of platelet-derived growth factor receptor (PDGFR), vascular endothelial growth factor receptor (VEGFR), rearrange during transfection (RET), and C-kit, is the first multi-kinase inhibitor proved to be beneficial in unresectable, advanced-stage HCC. The SHARP trial is the first phase III, placebo-controlled trial of the use of sorafenib in HCC. In this landmark study involving 602 patients with advanced disease naïve to systemic treatment, median OS was significantly improved in the sorafenib group compared to the placebo group (10.7 versus 7.9 months, *P* <0.001) ^25^. Another multi-national randomized controlled trial in the Asia-Pacific region, where chronic hepatitis B is the major risk factor for HCC, also confirmed the findings in the SHARP study ^26^. Further analysis of the two randomized controlled trials identified HCV-related HCC, absence of extrahepatic spread, and low neutrophil-to-lymphocyte ratio as predictors of greater survival benefit with sorafenib ^27^. The remarkable result with sorafenib is considered a major breakthrough in more than 30 years of pursuing a systemic treatment for HCC. However, the therapeutic window is narrow with sorafenib, with restrictions to patients with good performance status and compensated cirrhosis. Also, dose-limiting side effects including hand-foot-skin reaction, diarrhea, and weight loss are not uncommon. However, recent studies have shown an association between dermatological adverse effects with sorafenib with better treatment outcomes ^28, 29^. Over subsequent years, other agents have been studied as alternative first-line treatments against sorafenib or as second-line treatments against placebo in patients intolerant to or who have progressed while on sorafenib. Sunitinib, brivanib, linifanib, everolimus, and tivantinib were unable to meet their respective study end points as either non-inferior or superior to sorafenib or show survival benefit in those who failed sorafenib ^30– 34^.

Lenvatinib, an inhibitor of epidermal growth factor receptor (EGFR), fibroblast growth factor receptor (FGFR), VEGFR, PDGFR, RET, and C-kit, emerged almost a decade after sorafenib as an alternative first-line treatment for advanced HCC. Non-inferiority to sorafenib in terms of OS (13.6 versus 12.3 months, 95% confidence interval [CI] 0.79–1.06) was demonstrated in the REFLECT trial ^35^. In patients who progressed while on sorafenib, regorafenib and cabozantinib prolonged survival in their respective phase III randomized controlled trials ( Table 2). Bruix *et al*. demonstrated that regorafenib treatment resulted in longer median survival than placebo (10.6 versus 7.8 months; hazard ratio [HR] 0.63, *P* <0.001) ^36^. Comparable improvement in median OS was shown with cabozantinib by Abou-Alfa *et al*. (10.2 versus 8.0 months; HR 0.76, *P* = 0.005) ^37^. The recombinant IgG1 monoclonal antibody ramucirumab, which inhibits type 2 VEGFR-mediated angiogenesis, is the latest approved agent for advanced HCC. The initial REACH trial using ramucirumab failed to demonstrate benefit over placebo in patients with BCLC stage B and C disease not amenable to locoregional therapy and treated with first-line sorafenib ^38^. However, the effect of ramucirumab was noted to correlate with baseline AFP level, and benefit in OS was seen in the subgroup of patients with baseline AFP concentration above 400 ng/ml. The finding formed the basis of the follow-up REACH-2 trial, in which only sorafenib-treated patients with baseline serum AFP concentration over 400 ng/ml were recruited. Median OS (8.5 versus 7.3 months; HR 0.71, *P* = 0.020) and progression-free survival (PFS) (2.8 versus 1.6 months; HR 0.45, *P* <0.001) were significantly improved in the ramucirumab group compared with the placebo group ^39^. The study also highlighted that HCC is a heterogeneous disease and that stratification with biomarkers such as AFP is important in the future development of cancer therapy.

**Table 2.** Phase III trials on first- and second-line systemic therapy in hepatocellular carcinoma.

Trial name	Study drug	Control	No. of patients	Median overall survival (months)	Major side effects of study drug
**First line**					
SHARP	Sorafenib	Placebo	602	10.7 versus 7.9 HR 0.69, 95% CI 0.55–0.87, *p*<0.001	Diarrhea, weight loss, hand- foot-skin reaction, and hypophosphatemia
REFLECT	Lenvatinib	Sorafenib	954	13.6 versus 12.3 HR 0.92, 95% CI 0.79–1.06, non-inferiority met	Hypertension, diarrhea, decreased appetite, and decreased weight
IMbrave150	Atezolizumab plus bevacizumab	Sorafenib	501	Not estimated versus 13.2 HR 0.58, 95% CI 0.42–0.79, *p*<0.001	Hypertension, proteinuria, Increase AST, decrease platelet
Checkmate-459	Nivolumab	Sorafenib	743	16.4 versus 14.7 HR 0.85, 95% CI 0.72–1.0, *p*=0.0752	Not yet published
HIMALAYA	Durvalumab plus tremelimumab	Sorafenib	Ongoing	–	–
LEAP-002	Lenvatinib plus pembrolizumab	Lenvatinib	Ongoing	–	–
COSMIC-312	Cabozantinib plus atezolizumab	Sorafenib	Ongoing	–	–
ORIENT-32	Sintilimab plus IBI305	Sorafenib	Ongoing	–	–
NCT03764293	Apatinib plus SHR-1210	Sorafenib	Ongoing	–	–
**Second line**					
RESORCE	Regorafenib	Placebo	573	10.6 versus 7.8 HR 0.63, 95% CI 0.50–0.79, *p*<0.001	Hypertension, hand-foot-skin reaction, fatigue, diarrhea
CELESTIAL	Cabozantinib	Placebo	707	10.2 versus 8.0 HR 0.76, 95% CI 0.63–0.92, *p*=0.005	Palmar-plantar erythrodysesthesia, hypertension, increased AST, fatigue, diarrhea
REACH-2	Ramucirumab	Placebo	292	8.5 versus 7.3 HR 0.71, 95% CI 0.531–0.949, *p*=0.0199	Hypertension, hyponatremia, increased AST
KEYNOTE-240	Pembrolizumab	Placebo	413	13.9 versus 10.6 HR 0.781, 95% CI 0.611– 0.998, non-significant at prespecified threshold	Increased ALT/AST, increased bilirubin
KEYNOTE-394	Pembrolizumab	Placebo	Ongoing	–	–

AST, aspartate aminotransferase; ALT, alanine aminotransferase; CI, confidence interval; HR, hazard ratio.

## Immunotherapy

The past decade has witnessed major breakthroughs in cancer immunotherapy, which has demonstrated benefit in various solid organ and hematological malignancies ^40^. HCC develops in an inflammatory milieu, and immune tolerance is reported to play an important role in tumor pathogenesis ^41, 41^. Immune checkpoint inhibitors (ICPIs) targeting the programmed cell death protein 1/programmed death-ligand 1 (PD-1/PD-L1) pathway and cytotoxic T-lymphocyte-associated antigen 4 (CTLA-4) pathway are the two most studied immunotherapy mechanisms in HCC ( Figure 1). The two pathways are believed to act at different stages of the immune response. The PD-1/PD-L1 pathway promotes immune tolerance by suppressing the activity of T cells and mediating the differentiation of regulatory T cells, while the CTLA-4 pathway prevents autoimmunity by inhibiting the proliferation of potential autoreactive T cells ^43, 44^. By inhibiting these immune checkpoints, self-tolerance to malignant cells is lost, resulting in immune-mediated clearance of tumor tissue.

### CTLA-4 inhibitors

CTLA-4 blockage with tremelimumab is the first checkpoint inhibitor being evaluated as a potential treatment for hepatitis C-related HCC. A phase II study found that tremelimumab exhibits both anti-tumor and antiviral activities, with an acceptable safety profile ^45^.

### PD-1/PD-L1 inhibitors

Nivolumab is a monoclonal antibody inhibiting PD-1 receptor and demonstrated a favorable response rate of 20% in the dose expansion and escalation study (CheckMate 040) ^46^. The median OS in sorafenib-experienced patients in this phase II study was 16.7 months with an overall response rate of 14.5%. A similar response rate and improvements in OS, objective response rate, and complete response rate were confirmed in the subsequent head-to-head comparison against sorafenib as first-line therapy in advanced HCC (CheckMate 459), but the primary endpoint of OS did not reach statistical significance ^47^.

Pembrolizumab is another anti-PD-1 monoclonal antibody being investigated as a potential treatment for advanced HCC. In the phase II Keynote-224 trial, an objective response was observed in 18 of 104 sorafenib-experienced patients (17%), including one complete and 17 partial responses ^48^. A consistent result was seen in the Keynote-240 study, a follow-up phase III randomized controlled trial comparing pembrolizumab with best supportive care as second-line therapy after sorafenib, but the co-primary endpoint of improvement in OS and PFS was not met ^49^. A similar phase III study using pembrolizumab in Asian subjects is being conducted (Keynote-394, NCT03062358).

### CTLA-4 and PD-1/PD-L1 combination

Studies have also looked into a potential synergistic effect with combined inhibition of PD-1 and CTLA-4 pathways. Tremelimumab together with the anti-PD-L1 monoclonal antibody durvalumab have demonstrated favorable anti-tumor activity with acceptable tolerability in a small pilot study involving patients with advanced HCC or biliary tract carcinomas ^50^. A phase III multicenter randomized study (HIMALAYA, NCT03298451) is currently underway testing the dual therapy against sorafenib as first-line treatment in patients with unresectable HCC. The combination of nivolumab and the CTLA-4-inhibiting antibody ipilimumab also showed efficacy with a median OS of 23 months in its dose-finding study ^51^.

The side effect profile of ICPIs is diverse and distinctive from conventional cytotoxic chemotherapy. The immune-related adverse reactions can affect virtually any organ but most commonly involve the endocrine system, skin, gastrointestinal tract, and liver. For grade 1 toxicity, such as mild endocrinopathy, ICPIs can be continued with close monitoring and hormone replacement. Grade 2 or above toxicity usually mandates interruption of ICPIs with or without the need for steroids and immunosuppressants ^52^. Compared with patients with melanoma and non-small-cell lung cancer, HCC patients tend to have more elevation of AST/alanine aminotransferase when given ICPIs ^53^. It can be difficult to differentiate hepatitis associated with ICPIs from other causes of deranged liver function such as disease progression, reactivation of concurrent viral hepatitis, or decompensation of underlying cirrhosis.

## Combination of therapies of different mechanisms

Investigators have brought together locoregional therapy with systemic therapy or systemic therapy of different classes with the hope of augmenting tumor response. The initial experience with adding sorafenib or other molecular targeted therapy to TACE was disappointing ^54^. Inadequate dose and duration of sorafenib in these trials were identified as key reasons for failure. The Japanese TACTICS trial eventually demonstrated superiority of TACE plus sorafenib over TACE alone in terms of longer PFS (25.2 versus 13.5 months, *P* = 0.006) and longer time to TACE untreatable progression (26.7 versus 20.6 months, *P* = 0.020) ^22^. The SORAMIC study looked into the combination of sorafenib and SIRT with Y90 compared with sorafenib alone for advanced HCC. The primary endpoint of OS was not met, but subgroup analysis of non-cirrhotic patients or patients aged under 65 years did show a survival benefit with combination therapy ^23^. Future trials should address issues such as timing of sorafenib administration in relation to intra-arterial therapy, patient selection for such combination therapy by balancing potential benefits with incremental adverse effects, and more precise definition of both treatment efficacy and failure.

The combination of atezolizumab, an anti-PD-L1 antibody, and bevacizumab, an anti-VEGF antibody, has been investigated as a first-line systemic therapy in unresectable or metastatic HCC. It is postulated that the anti-angiogenic and immunomodulatory effect of bevacizumab can augment the anti-tumor immune activity of atezolizumab, as shown by a partial response rate of 62% in the phase I trial ^55^. Encouraging results were recently reported from the phase III IMbrave 150 trial comparing the dual therapy with sorafenib, in which statistically significant improvement in both OS and PFS was shown (HR of OS 0.58, *P* <0.001 and HR of PFS 0.59, *P* <0.001) ^56^.

Additional phase III trials on combinations of systemic therapies including lenvatinib and pembrolizumab (NCT03713593), cabozantinib and atezolizumab (NCT03755791), sintilimab and IBI305 (NCT0379440), and apatinib and SHR1210 (NCT03764293) are currently ongoing ( Table 2).

## Conclusion

HCC remains a deadly disease, and unresectable HCC is associated with limited survival. Considerable advancement in local and systemic therapies has been achieved in recent years owing to better understanding of the tumor microenvironment and the interplay between the tumor and the host immune system. With the latest breakthrough achieved with ICPIs, the armamentarium of treatment for HCC is rapidly expanding. However, heterogeneity in patient selection and definition of treatment response in the existing literature make the application of trial results to real-world practice difficult. With the standardization of trial design, prognostic markers of treatment response can be identified, allowing the selection of patients for the most appropriate anti-tumor therapy. Future studies should also continue to utilize therapeutic agents of different classes to achieve synergistic activity while minimizing toxicity.
